# Supplementary figures and images for: Automatic classification and prognosis prediction of cerebral hemorrhage based on a deep learning model
Source: Front Neurol. 2026 Feb 12;17:1725732. doi: 10.3389/fneur.2026.1725732 (PMC12937131; doi:10.3389/fneur.2026.1725732)

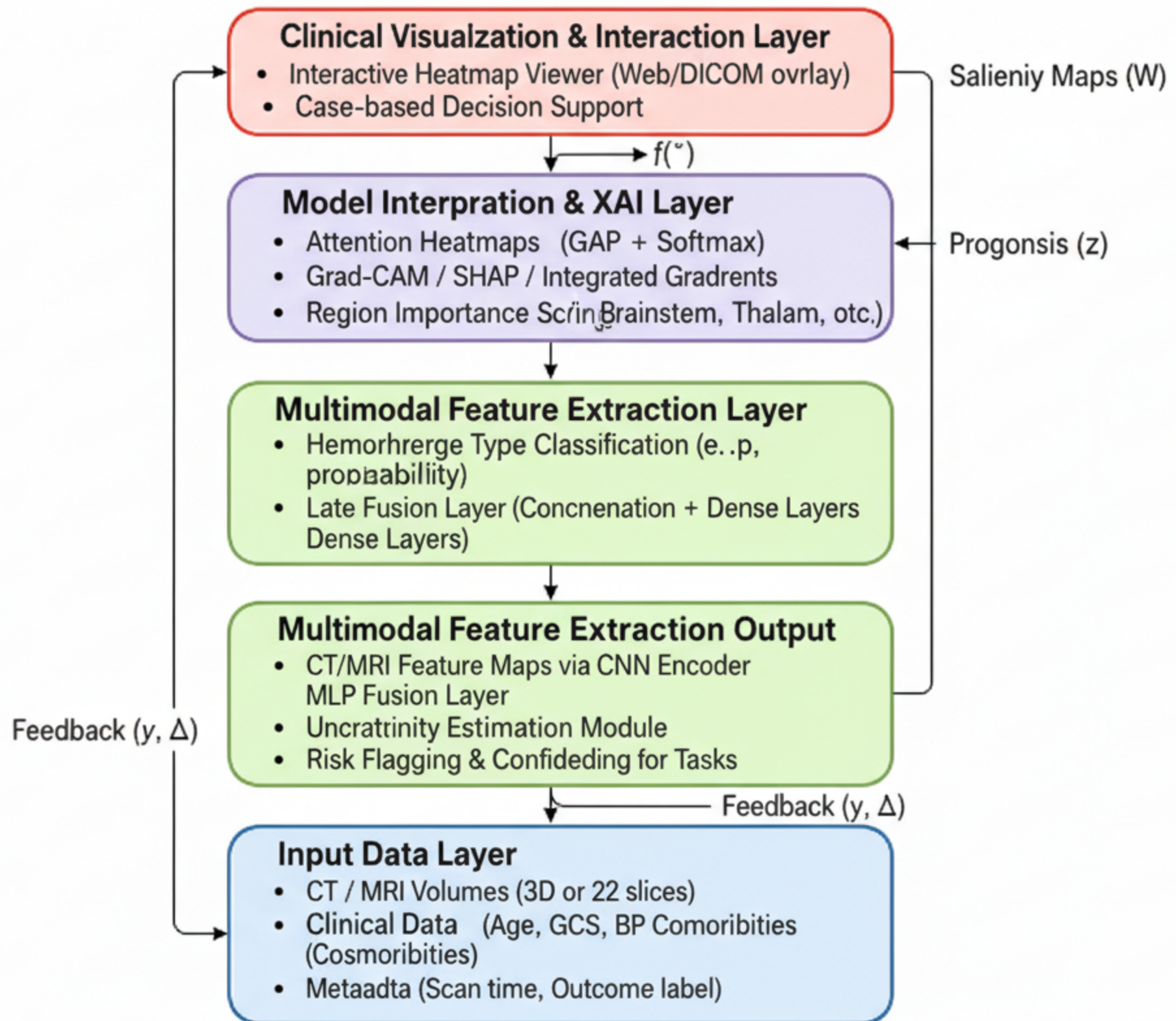

Supplement: Supplementary file 1 [file Data_Sheet_1.PDF]

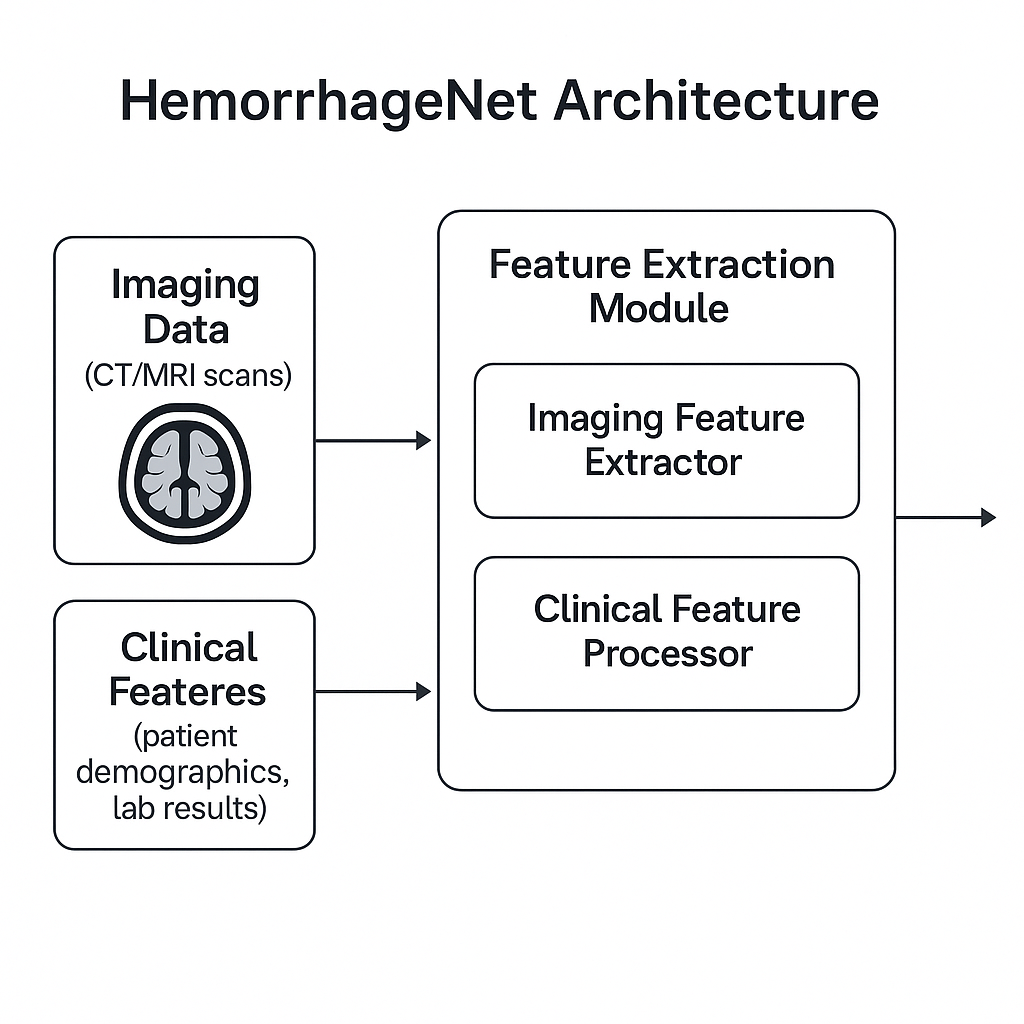

Supplement: Supplementary file 2 [file Image_1.PNG]

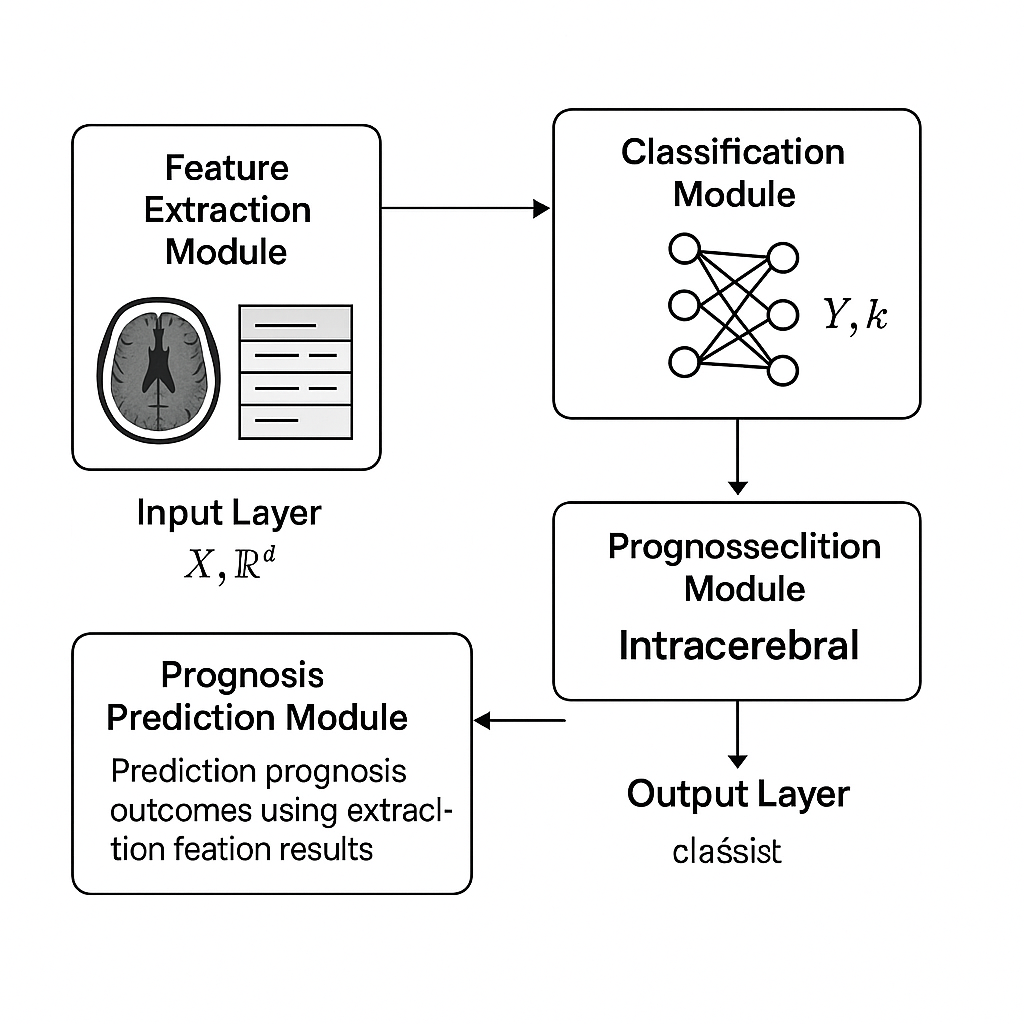

Supplement: Supplementary file 3 [file Image_2.PNG]

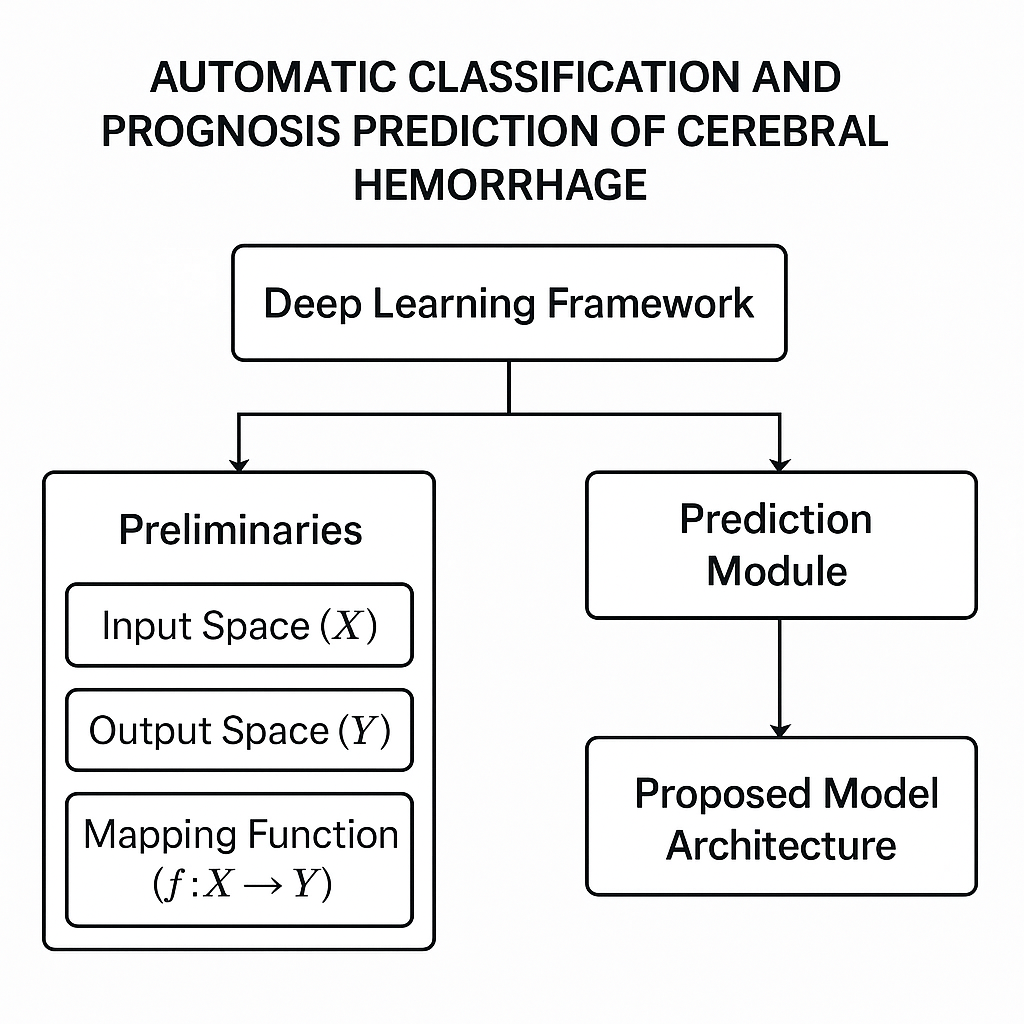

Supplement: Supplementary file 4 [file Image_3.PNG]

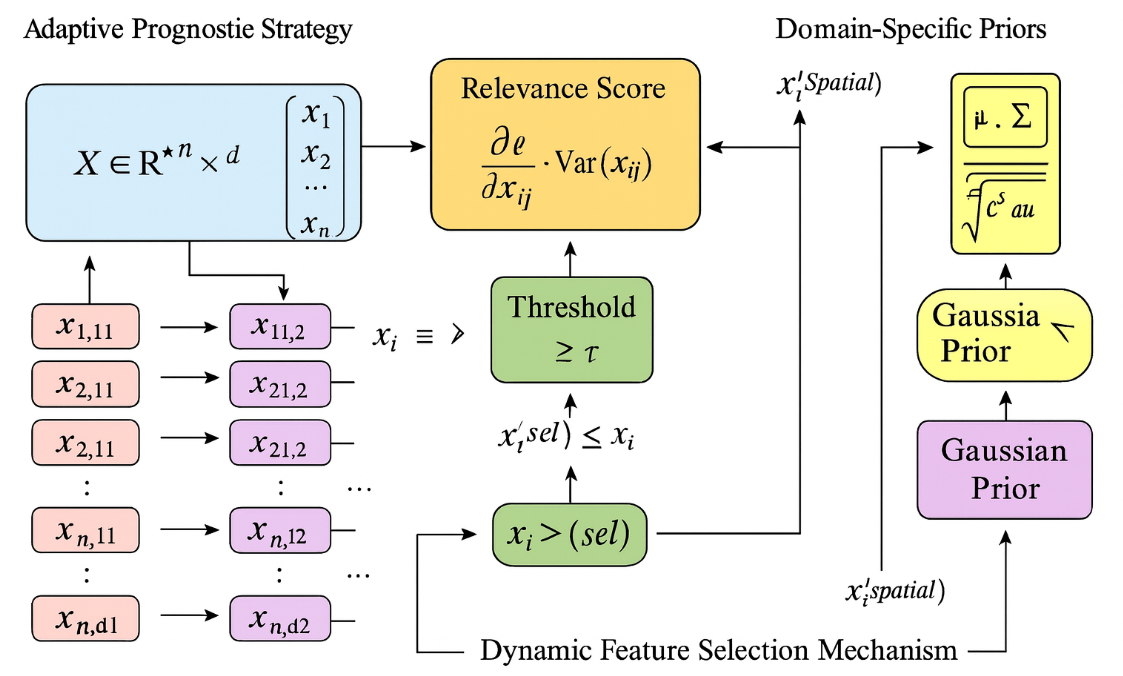

Supplement: Supplementary file 5 [file Image_4.PNG]
